# Discovery of Drug Candidates for Specific Human Disease Based on Natural Products of Gut Microbes

**DOI:** 10.3389/fmicb.2022.896740

**Published:** 2022-06-15

**Authors:** Cheng-Yu Wang, Qing-Feng Wen, Qiao-Qiao Wang, Xia Kuang, Chuan Dong, Zi-Xin Deng, Feng-Biao Guo

**Affiliations:** ^1^Department of Respiratory and Critical Care Medicine, Zhongnan Hospital of Wuhan University, Key Laboratory of Combinatorial Biosynthesis and Drug Discovery, Ministry of Education and School of Pharmaceutical Sciences, Wuhan University, Wuhan, China; ^2^School of Life Science and Technology, Center for Informational Biology, University of Electronic Science and Technology of China, Chengdu, China

**Keywords:** gut microbiota, natural products, microbial metabolites, human disease, drug discovery, IUC

## Abstract

The beneficial metabolites of the microbiome could be used as a tool for screening drugs that have the potential for the therapy of various human diseases. Narrowing down the range of beneficial metabolite candidates in specific diseases was primarily a key step for further validation in model organisms. Herein, we proposed a reasonable hypothesis that the metabolites existing commonly in multiple beneficial (or negatively associated) bacteria might have a high probability of being effective drug candidates for specific diseases. According to this hypothesis, we screened metabolites associated with seven human diseases. For type I diabetes, 45 out of 88 screened metabolites had been reported as potential drugs in the literature. Meanwhile, 18 of these metabolites were specific to type I diabetes. Additionally, metabolite correlation could reflect disease relationships in some sense. Our results have demonstrated the potential of bioinformatics mining gut microbes' metabolites as drug candidates based on reported numerous microbe-disease associations and the Virtual Metabolic Human database. More subtle methods would be developed to ensure more accurate predictions.

## Introduction

Microbes, whose population may reach 1,014 orders of magnitude (Thursby and Juge, 2017), dwell in the human gut since the emergence of the Homo species. The large population of microbes plays a critical and indispensable role in the life process, healthy balance, and disease development of their host species (Zhang et al., 2015; Cani, 2018; Gomes et al., 2018). Natural products from various sources had been explored as potential drug candidates in recent decades (Adegboye et al., 2021; Klunemann et al., 2021). With respect to the selection of natural products, scientists mainly performed their analysis depending on three traditional strategies that contain culture-based, (meta) genomics-based, and metabolomics-based approaches (Wang et al., 2019). These methods were excellent in digging into the valuable information produced in human gut microbiota, particularly when we combined all of them together (Wang et al., 2019). However, there is still a lack of bioinformatics methods to mine disease-specific metabolites from mixed bacterial species. Aiming to this, here, we developed a theoretical framework to explore the relationship between microbial natural products and human diseases and identified the potential drug candidates directly against corresponding diseases among microbial natural products.

The composition of gut microbiota keeps dynamic equilibrium since species enter their adult stage (Rodriguez et al., 2015). Otherwise, the breakdown of the dynamic equilibrium of microbiota would induce the corresponding disease (Makki et al., 2018). Thus, one potential treatment strategy is supplementing the microbes which have been lacking, such as supplementing probiotics and fecal microbiota transplantation (FMT) (Sokol et al., 2020; Snigdha et al., 2021). To some degree, we assumed not only the microbiota could exert influence on human diseases but also the microbial natural products did as well. To put it another way, the gut microbiota might not be used as a direct treatment, because productions from microbiota were extremely complex and the transplantation of gut microbiota often was not operable. The natural products of the supplied microbiota would be the bona fide ones that played roles in ameliorating human disease (Katz and Baltz, 2016; Brown and Hazen, 2017; Das et al., 2020). Based on this, we suggested that metabolites, which missing or reduced during the development of disease, could serve as potential drug candidates.

Ma et al. constructed a database named Human Microbe-Disease Association Database (HMDAD), which was an excellent knowledgebase gathering of the relationship between human diseases and gut microbes (Ma et al., 2017). Because many gut microbes are not cultivatable in the laboratory, some kinds of real natural products of microbes could not get through the experiment until now [8]. Fortunately, the Virtual Metabolic Human database (VMH) collected information on microbial metabolites from hundreds of manually curated genome-scale metabolic models based on genomic, biochemical, and physiological data (Magnusdottir et al., 2017; Noronha et al., 2019). We used these microbial metabolites as the natural products of these microbes. Additionally, we searched literature works for more related information between human diseases and gut microbes in PubMed to further validate our results.

Our work built the association of metabolite disease and proposed the “Intersection-Union-Complement (IUC)” method to screen the beneficial metabolites as drug candidates. As a primary key step before traditional experiments, this method can not only effectively identify the potential drug candidates for specific diseases from microbial metabolites but also greatly save the time and cost of researchers.

## Materials and Methods

### Data Obtained

From the HMDAD database http://www.cuilab.cn/hmdad (Ma et al., 2017) and literature, we obtained microbe information presenting increased or decreased status in the development of specific diseases (Supplementary Table S1). To search for specific natural products of the disease, information on gut microbiota population change is necessary. We obtained 7 common diseases and their relevant microbes whose metabolites information was found in the VMH database. It should be noted that the information of microbes in VMH was at the strains resolution level, while the information in HMDAD was at the species resolution level. Thus, the natural product information of one microbial species came from the metabolite intersection set of all strains belonging to the same species in VMH (Noronha et al., 2019).

### Intersection-Union-Complement Method

**Figure 1:**
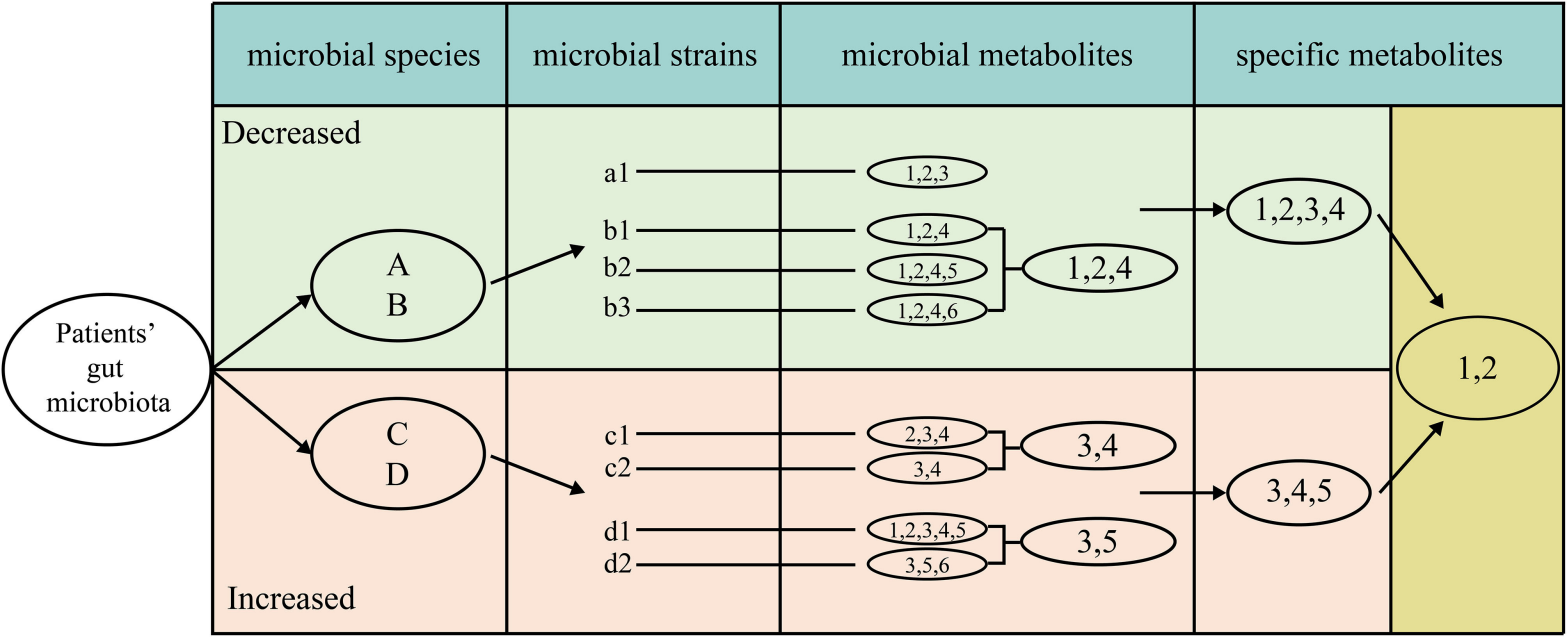
The detailed process of screening metabolites specific for one disease, which as four steps, extracting metabolites for each strain, intersecting the metabolites among different strains in each species, uniting the metabolites for all beneficial and all detrimental microbes, subtracting the union of beneficial metabolites to detrimental metabolites.

For screening the specific natural products of one disease, we developed an IUC method which was described below (Figure 1). The IUC method could be divided into four steps in detail: (a) we gathered disease-microbe association information, most of which was at the species resolution level; if the literature reported the association at the genus level, then we assume that all the species within it have the same association property. (b) VMH database contained many manually curated genome-scale metabolic information. Some species might have different strains, which are represented by relevant strains in the VMH database. We used a lower-case alphabet with a number behind it to represent the strain that belonged to the corresponding species represented by an upper-case alphabet. For example, strain “a1” belongs to the “A” species. For the different strains of one specific species, we took the intersection set of these strains' metabolites as the natural products for that species. For instance, “1,” “2,” and “4” were the intersection of metabolites of three strains “b1,” “b2,” and “b3,” thus we took these metabolites as the natural products of species “B.” (c) Each microbial species had its metabolites, while the union metabolites set of all decreased microbial species were taken as the metabolites of decreased microbes, the same method was used to increased microbes. (d) We assumed that the metabolites which appeared only in decreased microbes were tended to be beneficial when given a specific disease. That was to say, we took the method that the union metabolites set of decreased microbes subtracted the union part of increased microbes, the results of which were taken as the specific natural products for one disease.

## Results

### The Microbial Metabolites of Specific Diseases Screened by the Intersection-Union-Complement Method

We assumed that natural products from decreased gut microbiota were beneficial to specific human diseases, while the ones from increased gut microbiota were harmful to that disease. Based on this idea, we developed an “intersection-union-complement (IUC)” method (see in the “Method” section) to screen the specific beneficial natural products associated with specific diseases.

Depending on the disease-microbe information we initially gathered, we modeled disease-metabolites association based on the methodology of IUC. If one microbe had been reported to have conflicting information of associations with one specific disease, that is, when literature A assumes it is deceased whereas literature B is increased compared with a healthy status, then we neglected this microbe. We totally obtained 1,024 disease-metabolites associations which contained 691 unique metabolites from 7 diseases (Table 1). The 691 unique metabolites could be divided into several categories such as cholic acid, vitamin derivative, indole derivative, intermediates in fatty acid metabolism, and so on. For example, colorectal carcinoma had an increased microbe (Lactobacillus casei Bl23) and a decreased microbe (Fusobacterium nucleatum) which came from 2 references, and the number of colorectal carcinomas specific natural products was 254 according to our method (Table 1). We assume that the core metabolites of common microbes could be filtered from the result by our fourth step operation of subtracting. Furthermore, our result is independent of species abundance distribution with each metagenomic study and only dependent on the property spectrum of species-disease association. We also tried the more rigorous methods by using the intersection of decreased microbes and subtracting the union of increased microbes. By this method, there were no beneficial metabolites screened for Crohn's disease, liver cirrhosis, and type 1 diabetes (Supplementary Table S2). This suggested the large potential beneficial metabolites were lost by using overly rigorous methods.

**Table 1:** The general information on diseases, microbes, and natural products.

**Disease**	**Number of increased microbes**	**Number of decreased microbes**	**Number of specific natural products**	**Number of references**
Colorectal carcinoma	1	1	254	2
Crohn's disease	4	8	166	6
Irritable bowel syndrome	1	4	229	4
Liver cirrhosis	11	14	121	2
Necrotizing enterocolitis	1	2	41	1
Obesity	2	3	125	5
Type 1 diabetes	6	6	88	4

### Quantifying the Similarities Between Diseases Based on Shared Metabolites

**Figure 2:**
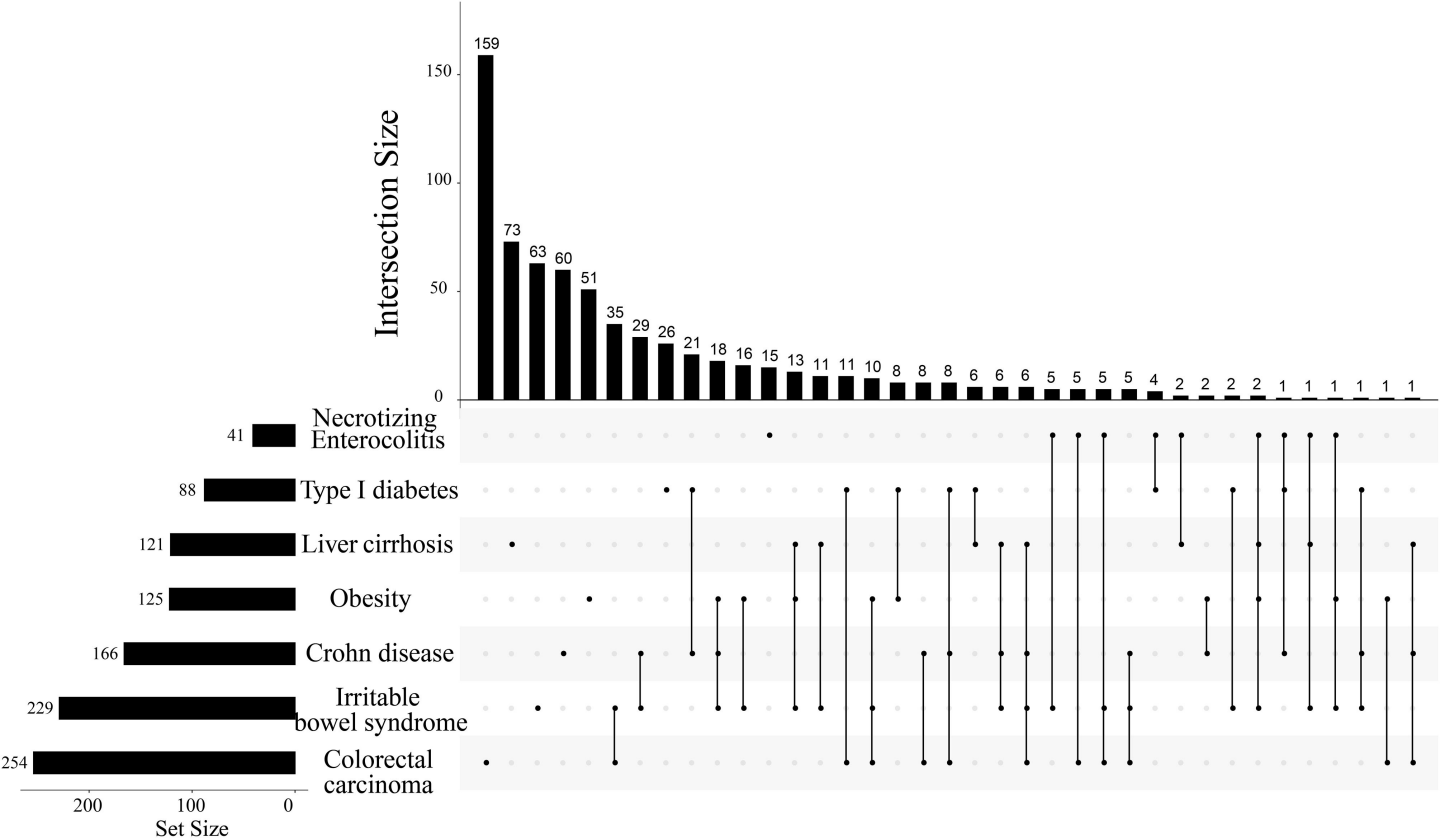
The intersection size of natural products among 7 different diseases. The top number denotes the intersection size of diseases marked by bold folds on the line in the right lower part. For example, according to the order from left to the right, the first isolated point denotes that colorectal carcinoma has a total of 254 identified metabolites whereas it has 159 metabolites. The first line has two marked points and they meant that colorectal carcinoma and irritable bowel syndrome have 159 common metabolites beneficial to both diseases. The largest points marked on one line are four and it corresponds to the 24th line. This line means that the marked four diseases have only two common metabolites beneficial to all of them.

Based on the list of specific natural products of seven diseases, we found that metabolites repeatedly presented in multi-species, which might be associated with more than two diseases. We assumed that the more metabolites shared by the diseased, the more similarities presented between the two diseases. Hence, we were interested in whether the screened metabolites could reflect the similarities between diseases or not (Table 1), at least giving a basic profile. To explore the disease-disease relationships and validate this conjecture, we counted the common natural products of different diseases. If some different diseases shared a few common natural products, it may imply common symptoms or pathogenesis between these diseases (Figure 2). As shown, even as many as four different diseases shared the same two metabolites.

**Figure 3:**
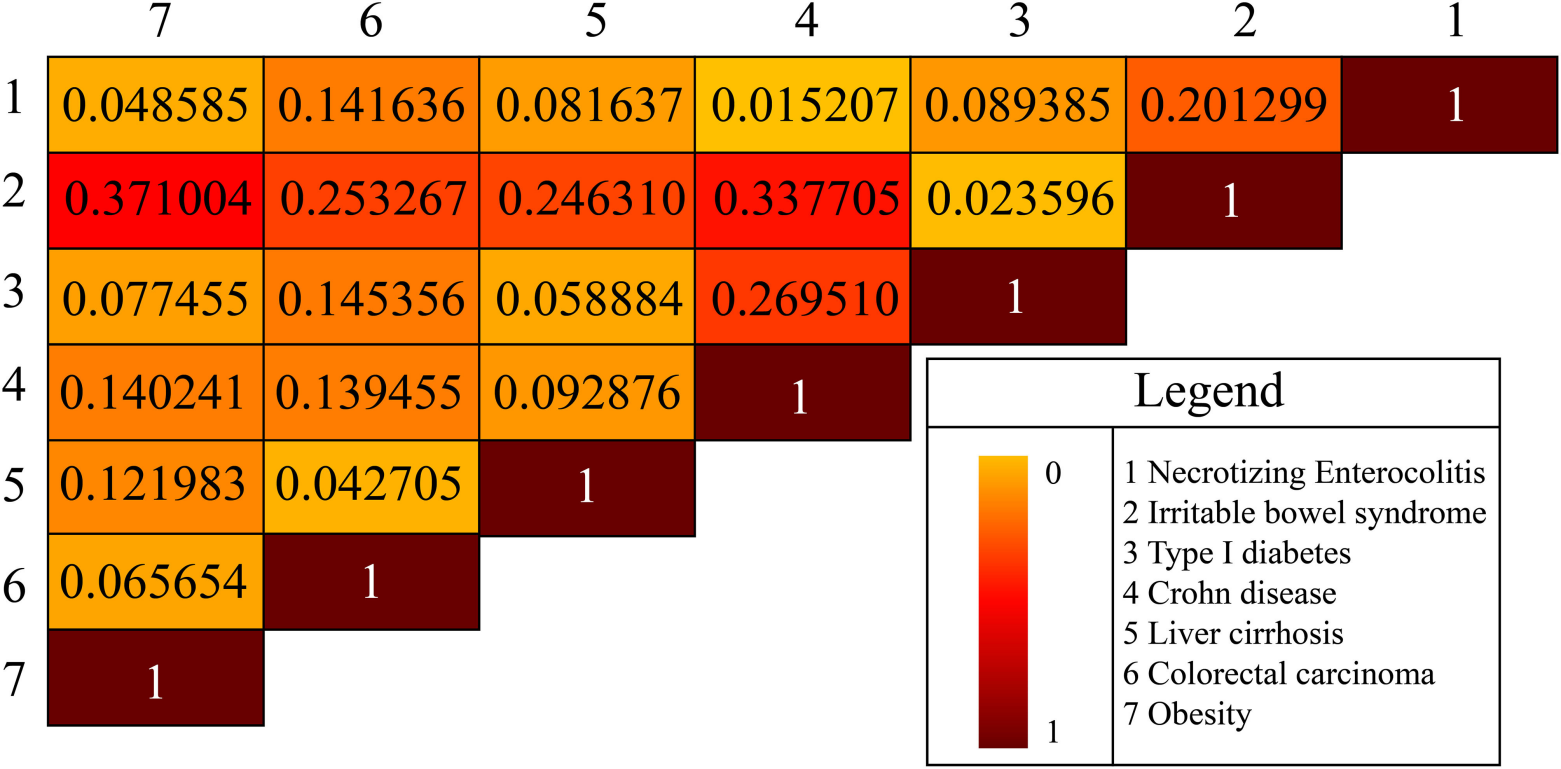
The disease-disease association is based on microbial natural products. There are seven diseases in total.

Background noise might be introduced manually when we simply used absolute metabolite numbers from the gut microbiota to measure similarity or association between two diseases. Hence, we used the average of the two ratios of the common metabolites to the respective metabolites in either disease to denote the similarity of the two disease types. Based on the above idea and the specific microbial natural products of each disease, we constructed a heat map to exhibit the similarity between pairwise diseases (Figure 3). For example, obesity and irritable bowel syndrome (IBS) shared 60 common microbial natural products, and the similarity between the two diseases was calculated *via* the proportion of common natural products in obesity and in IBS, which was (60/125 + 60/229) ^*^1/2 = 0.371 and ranged in top 1 compared with the similarity score between obesity and other diseases. Meanwhile, some literature works suggested that IBS was more common in patients with morbid obesity than in the general population, with a prevalence rate ranging from 8 to 31% in small series (Fysekidis et al., 2012; Santonicola et al., 2013; Bouchoucha et al., 2015, 2016; Schneck et al., 2016). For the heterogeneity in the different samples of populations, it remained unclear whether IBS was associated with obesity (Pickett-Blakely, 2014). Scientists proposed that gut microbiota transfer might explain a possible connection between obesity and IBS (Ley et al., 2005, 2006; Kassinen et al., 2007; Turnbaugh et al., 2008; Rajilic-Stojanovic et al., 2011; Jeffery et al., 2012; Pickett-Blakely, 2014). A study illustrated that a potential mechanism whereby IBS symptoms manifested in obese persons might be induced by small intestinal bacterial overgrowth (Madrid et al., 2011). Accordingly, we achieved the same conclusion (association relationship between IBS and obesity) *via* constructing the comparisons of metabolites shared by different diseases. Our results also demonstrated that IBS and Crohn's (IBS vs. Crohn's) showed a comparable similarity score (0.3377, the second-highest one) compared with IBS vs. obesity. A recent study reported IBS and Crohn's disease shared some common symptoms which presented a clinical dilemma for physicians (Halpin and Ford, 2012). Therefore, we may get the conclusion cross-disease relationship may be reflected by the accordance of their metabolites.

### The Relationship Between Metabolites and Diseases Is Steady

Several drawbacks could influence our conclusion; on the one hand, the information between diseases and microbes was incomplete, while on the other hand, there might be imperfect matching between microbial species and microbial natural products in the VMH database. So, we wondered whether the association between microbial natural products and diseases remained stable or not when the two imperfect conditions existed in our collected data. To check the stability of the disease-disease association, we randomly deleted two disease-associated microbes from liver cirrhosis, since the number of increased microbes and decreased microbes in liver cirrhosis was relatively larger. We made 100 times of random deletion operations, and the associations of Liver cirrhosis with other diseases were shown in Supplementary Figure S1. Compared with previous results, the similarity difference of liver cirrhosis with other 6 diseases differed from 0 to 0.076, which meant a small difference between the two results. Even if we randomly deleted two microbes, not only natural products but also disease-disease associations were nearly the same in the results. This observation also supported our conclusion that the diseases might have a similar regulatory mechanism which is reflected by metabolites of gut microbiota.

### Screened Products From Microbial Are Potential Drug Candidates

Among different diseases, the count of metabolites ranged from 41 to 254 (Supplementary Table S3) spanning a relatively large scope, which led the selection of the potential drug from natural product candidates difficult. Since the information we gathered was insufficient, these natural products might not be totally ideal specific to one disease. Here, we took Type 1 diabetes as a case to exhibit to which extent the screened metabolites could be regarded as drug candidates.

With the four steps procedure, there were 88 selected metabolites specific for Type 1 diabetes, and 62 of the 88 metabolites were shared with other 6 diseases. We searched for more information on these chemical substances in PubChem (Kim et al., 2019) (Supplementary Table S4). Among which, 45 metabolites were supported with disease-related by literature works, and 18 metabolites of 45 were Type 1 diabetes-related. Thus, we searched for more literature works for the 18 metabolites in PubMed.

As we scrutinized literature works, a microbial natural product named Phytoene, i.e., a kind of carotenoid, was found to be able to activate the antioxidant response (Ley et al., 2006). When combined with antihyperglycemic agents, carotenoids demonstrated the potential superior protection against cellular oxidative injury in diabetes (Kassinen et al., 2007). This was because the autoimmune-mediated destruction of pancreatic beta-cells in type 1 diabetic patients also involved a phase of oxidative stress (Turnbaugh et al., 2008; Rajilic-Stojanovic et al., 2011). 3-Hydroxy-3-methylglutaryl coenzyme A (HMG-CoA) participated in the cholesterol metabolism as important stuff. HMG-CoA reductase inhibitors (statins) reduced activation of the transcription factor NF-κB (Oda and Keane, 1999), while the redox-sensitive transcription factor nuclear factor NF-κB was believed to contribute to late diabetic complications (Bierhaus et al., 1997). So, we assumed part of these selected natural products might be the potential drug in the treatment of diabetes.

As to the other part of 43 metabolites that were not related to diabetes, we proposed that even in these products there were some potential drug candidates for diabetes. Here, we chose the natural product Protoporphyrin IX as a case study. In Hana Farhangkhoee et al.,' study, Protoporphyrin IX was utilized as a potential inhibitor of the heme oxygenase system (Drummond and Kappas, 1981) to treat streptozotocin-induced diabetic rats (Farhangkhoee et al., 2003). The author stated that the heme oxygenase system was responsible for increased oxidative stress, which was involved in cellular damage and diabetic cardiomyopathy. Therefore, Protoporphyrin IX had the potential to restrain diabetes during the progression of cardiomyopathy.

To sum up, due to the insufficient disease-microbe information that we gathered and imperfect matching between microbes and natural products, the number of final chosen metabolites may be still quite large. However, we have shown that most identified metabolites have literature supporting associations for Type 1 diabetes. We know that not all of the identified could be validated as drugs, but compared with the original number of metabolites involved in this work (Table 1), we assume that our screening procedure got a satisfactory result by reducing folds. These metabolites that have no literature proof need to be further investigated using cell or animal models.

## Discussion and Conclusion

Metabolites had been used as drugs for a long time (Saha et al., 2016; Dvorak et al., 2020). Gut microbes can produce thousands of metabolites exerting impactions on human health (Zhang et al., 2015; Hernandez et al., 2019). Therefore, we assumed the metabolites of gut microbes could also be used as candidate drugs. Because of the wide range of metabolites, verifying the effect of all these metabolites in animal models was costly and unrealistic. Therefore, we proposed a theoretical drug research method to screen the metabolites by performing a series of operations of intersecting, uniting, and complementing.

This procedure could efficiently narrow down the range of metabolite candidates through the theoretical method of common metabolites from various beneficial bacteria. In our present work, we performed screening based on the virtual metabolites. The accuracy of our method would be further improved by substituting these virtual metabolites using the actual metabolites for beneficial bacteria by metabolome analysis when available. The qualitative IUC is a simple method to screen gut microbes metabolites associated with specific diseases. Because of the simplicity, it would be simply grasped and understood. However, it may not generate perfect predictions and we will improve it using the quantitative form in the future. Here, the method is only used to illustrate the potential of selecting drug candidates from gut microbes metabolites.

The matter biosynthesized or metabolized by gut microbes is really a biological treasure. These products are naturally existing in the human gut (Kadosh et al., 2020; Wang et al., 2020) and the toxic and side effects of these products as drugs would be reduced to the minimum when the dosage is controlled properly. The wet-lab-based mining method for these natural products may be time-consuming. There are so many microbe-disease association researches with high quality and they constitute the basis of our method when combined with the VMH database. More subtle methods need to be developed to obtain more accurate predictions.

## Data Availability Statement

The original contributions presented in the study are included in the article/Supplementary Material, further inquiries can be directed to the corresponding author/s.

## Author Contributions

F-BG conceived and guided the research study. C-YW and Q-FW completed data processing, analysis, and drafting of the paper. Q-FW, XK, and CD extracted data and visualized the results. C-YW and Q-QW wrote the manuscript. All authors reviewed, revised, and approved the final manuscript.

## Funding

This study was supported by the National Natural Science foundation of China (31871335) and by the fellowship of China's Postdoctoral Science foundation (2021M692476 and 2021TQ0254).

## Conflict of Interest

The authors declare that the research was conducted in the absence of any commercial or financial relationships that could be construed as a potential conflict of interest.

## Publisher's Note

All claims expressed in this article are solely those of the authors and do not necessarily represent those of their affiliated organizations, or those of the publisher, the editors and the reviewers. Any product that may be evaluated in this article, or claim that may be made by its manufacturer, is not guaranteed or endorsed by the publisher.

## Supplementary Material

Supplementary Figure S1 The disease-disease association is based on microbial natural products in the case of random deletion of two disease-associated microbes in Liver cirrhosis. The number in the bracket denotes the standard deviation for the 100 times of random deletion.

Supplementary Table S1 The count of natural products varied in different diseases.

Supplementary Table S2 The natural products of different diseases are screened by the opposite method.

Supplementary Table S3 Details of 88 selected natural products in Type 1 diabetes.

Supplementary Table S4 The quantity of statistics information on microbes in specific diseases.
