# Supplementary figures and images for: Discovery of Drug Candidates for Specific Human Disease Based on Natural Products of Gut Microbes
Source: Front Microbiol. 2022 Jun 15;13:896740. doi: 10.3389/fmicb.2022.896740 (PMC9240467; doi:10.3389/fmicb.2022.896740)

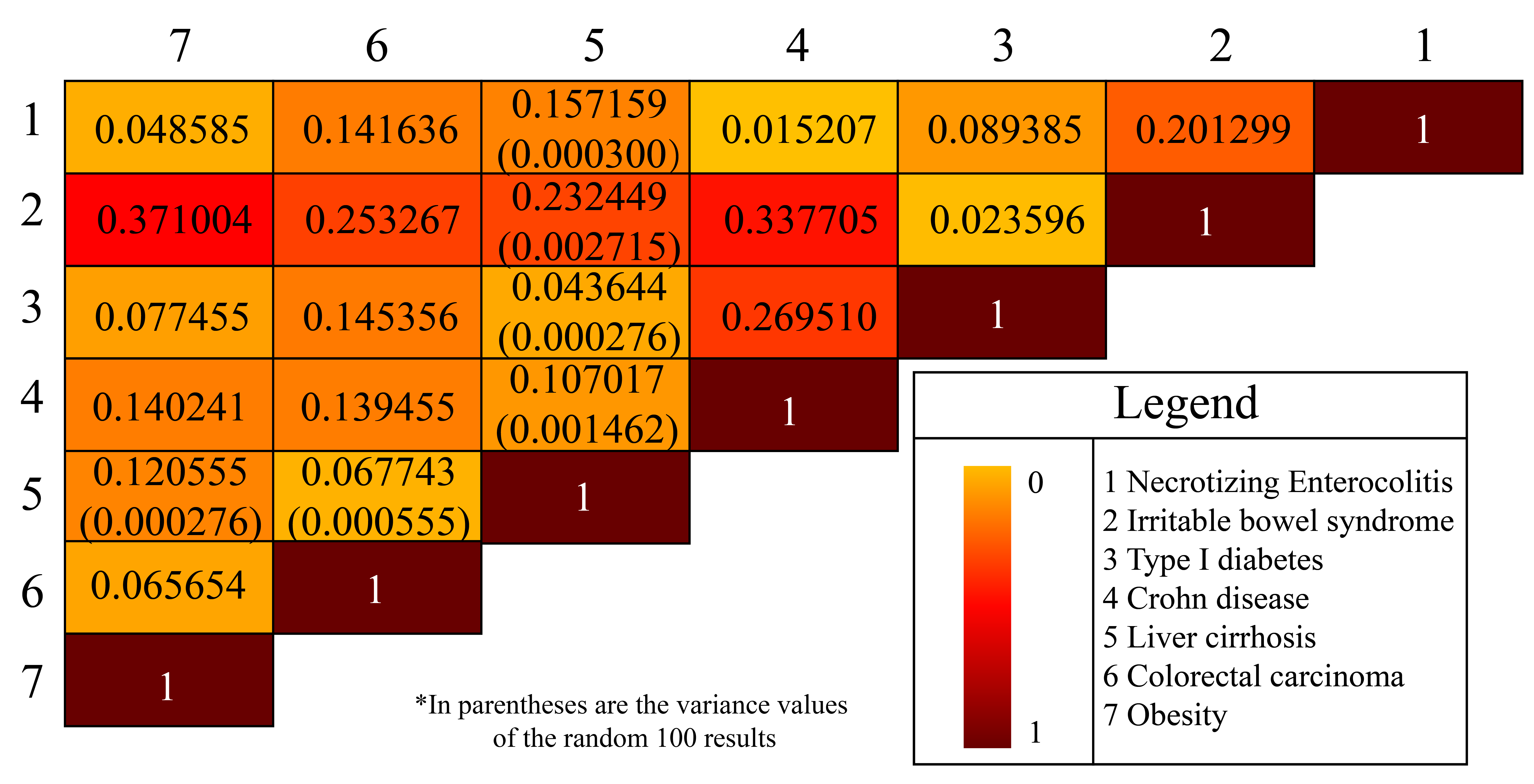

Supplement: Supplementary Figure S1 — The disease-disease association is based on microbial natural products in the case of random deletion of two disease-associated microbes in Liver cirrhosis. The number in the bracket denotes the standard deviation for the 100 times of random deletion. [file Image_1.TIF]
